# Suhuai suckling piglet hindgut microbiome-metabolome responses to different dietary copper levels

**DOI:** 10.1007/s00253-018-9533-0

**Published:** 2018-12-07

**Authors:** Feng Zhang, Weijiang Zheng, Yongqiang Xue, Wen Yao

**Affiliations:** 1 Jiangsu Key Laboratory of Gastrointestinal Nutrition and Animal Health, College of Animal Science and Technology, Nanjing Agricultural University, Nanjing, China; 2 College of Agriculture, Guangdong Ocean University, Zhanjiang, China; 3 Key Lab of Animal Physiology and Biochemistry, Ministry of Agriculture, Nanjing, China

**Keywords:** Piglet, Microbiota, Metabolite profiles, Copper, Hindgut

## Abstract

Unabsorbed copper accumulates in the hindgut of pigs that consume high levels of dietary copper, which enhances the coselection of antibiotic-resistant bacteria and is considered detrimental to the environment and to porcine health. In our study, a combination of 16S rRNA pyrosequencing and nontargeted metabolomics was used to investigate the microbiome-metabolome responses to dietary copper levels in the hindgut of suckling piglets. The results showed that the dietary copper level affected the abundance of several *Clostridia* genera and that the relative abundance of butyrate-producing bacteria, such as *Coprococcus*, *Roseburia*, and *Acidaminococcus*, was reduced in the 300 mg kg^−1^ (high) Cu group. Metabolomic analysis revealed that dietary copper levels affected protein and carbohydrate metabolites, protein biosynthesis, the urea cycle, galactose metabolism, gluconeogenesis, and amino acid metabolism (including the metabolism of arginine, proline, β-alanine, phenylalanine, tyrosine, and methionine). Furthermore, Pearson’s correlation analysis showed that the abundance levels of *Coprococcus* (family *Lachnospiraceae*) and operational taxonomic unit (OTU) 18 (family *Ruminococcaceae*) were positively correlated with energy metabolism pathways (gluconeogenesis, glycolysis, and the pentose phosphate pathway). The abundance of *Streptococcus* was negatively correlated with amino acid metabolism pathways (protein biosynthesis, glycine, serine, threonine, methionine, phenylalanine, and tyrosine metabolism), and OTU583 and OTU1067 (family *Rikenellaceae*) were positively correlated with amino acid metabolism pathways. These results suggest that the copper levels consumed by LC (low-copper group) versus HC (high-copper group) animals alter the composition of the gut microbiota and modulate microbial metabolic pathways, which may further affect the health of suckling piglets.

**Electronic supplementary material:**

The online version of this article (10.1007/s00253-018-9533-0) contains supplementary material, which is available to authorized users.

## Introduction

Copper is an essential microelement for life, and copper toxicity can occur in any species, including ruminants and monogastric animals, that consumes excessive amounts of supplemental copper. When ruminants consume too much copper, they accumulate copper in the liver before the toxicity becomes apparent. Stress or other factors can cause a large amount of copper to be suddenly released from the liver into the blood, leading to a hemolytic crisis. This type of crisis is characterized by considerable hemolysis, jaundice, methemoglobinemia, hemoglobinuria, systemic jaundice, extensive necrosis, and frequent death (Goff 2004; Puig and Thiele 2002; Scheiber et al. 2014). However, ruminants are much more susceptible than monogastric animals. Pigs can tolerate more than 250 mg kg^−1^ copper in their diet, and dietary copper at a level of 100 to 250 mg kg^−1^ has been widely reported to promote growth and increase the feed intake and growth performance of pigs (Armstrong et al. 2004; Fry et al. 2012; Huang et al. 2015; Lu et al. 2010; Zhou et al. 1994); 250 mg kg^−1^ dietary copper was considered the most efficacious level in nursery pigs (Ma et al. 2015). Therefore, feed is generally supplemented with a high concentration of copper in China’s swine industry.

In addition, many studies reported that dietary high levels of copper, used as an alternative to antibiotic growth promoters in animal production, might further enhance the coselection of antibiotic-resistant bacteria (Baker-Austin et al. 2006; Brown et al. 1992; Hasman and Aarestrup 2002; Hölzel et al. 2012; Seiler and Berendonk 2012). Excess copper in feed is considered to be detrimental to the environment via excretion whether or not the animal absorbs the copper (Armstrong et al. 2004; Jondreville et al. 2003; Veum et al. 2004). Thus, the question of whether excess supplementation of Cu in feed affects pig health is gaining renewed attention.

Our previous studies showed that high levels of copper in the diet lead to a major accumulation of unabsorbed copper in rat feces, which affects microbial composition by altering the populations of certain bacteria (Zhang et al. 2017a). The effect of dietary copper level on the gut microbiota of pigs is also complex and changeable. The addition of either bound (36 to 47 mg kg^−1^, Cu-montmorillonite) or inorganic (200 mg kg^−1^, CuSO_4_/CuO) copper has been reported to decrease the *Clostridium* population in piglets and growing pigs (Song et al. 2013; Xia et al. 2005). Dietary copper > 100 mg kg^−1^ (CuSO_4_) has been shown to reduce the *Streptococcus* population in the porcine gut but does not appear to have any effect on the coliform bacteria population (Bunch et al. 1961; Hawbaker et al. 1961; Hojberg et al. 2005; Kellogg et al. 1966; Mei et al. 2010; Namkung et al. 2006; Varel et al. 1987). Supplementation of > 170 mg kg^−1^ copper (CuSO_4_) in diets reduced *Enterococcus* and lactic acid bacteria populations in the stomach content, as well as *Lactobacillus* in the gastrointestinal tract of pigs; however, Cu in the form of CuO did not have the same effect (Hawbaker et al. 1961; Hojberg et al. 2005; Hu et al. 2004; Mei et al. 2010; Namkung et al. 2006; Wang et al. 2012; Xia et al. 2005). No effect on the anaerobic population was observed when the dietary copper level was < 200 mg kg^−1^ (Bunch et al. 1961; Hojberg et al. 2005; Mei et al. 2010; Varel et al. 1987). Interestingly, 250 mg kg^−1^ dietary Cu in the form of CuSO_4_ may reduce the total population of anaerobic bacteria in the colon, while CuO does not seem to have any effect even at 375 mg kg^−1^ Cu (Hawbaker et al. 1961; Hojberg et al. 2005).

There is a growing interest in gut microbiota and human health, and pigs are considered to be ideal models for human nutrition research (Guilloteau et al. 2010). Our previous studies suggested a potential link between dietary copper levels and rat health; unabsorbed copper accumulated in the gut, altering the composition of its microbiome and subsequently inducing an inflammatory response. Tumor necrosis factor (TNF)-α may be the chief molecule that responds to microbiotic shifts under excessive copper exposure (Zhang et al. 2017a). Taken together, these findings confirm that high levels of dietary copper change the composition of the microflora, which may further alter its metabolic activities and affect porcine health. However, to date, information regarding the effects of microbiome-metabolome responses to dietary copper levels in the hindgut of piglets is limited.

In the current study, 16S rRNA pyrosequencing and nontargeted metabolomic methods were designed and used to investigate the effect of dietary copper levels on microbiome-metabolome responses in piglets. This study evaluated the use of high levels of copper in the diet and explored the effect of copper level on the health of Suhuai suckling piglets to assess the safety and necessity of high levels of copper in feed.

## Materials and methods

### Ethical approval

This study was approved and implemented under the supervision of the Animal Protection and Utilization Committee of Nanjing Agricultural University (Nanjing, Jiangsu province, China). According to the Nanjing Agricultural University Animal Care and Use Guide, all pigs were raised on local commercial farms.

### Animals, housing, diets, and sampling

Suhuai suckling piglets were used as the experimental model in this study. This breed was developed via hybridization of a local Huai sow with a Yorkshire pig in 1958, followed by decades of breeding. Suhuai pigs were approved as a new breed by the China National Commission of Animal Genetic Resources in 2011.

One hundred seventy-two piglets from 18 multiparous Suhuai sows (inseminated twice) were sorted into blocks by their anticipated farrowing dates and assigned to two rooms (nine litters/room, 10 piglets per litter). All piglets were individually weighed at 72 h postnatally. Within each litter, all piglets were chosen based on similar body weight (BW); BW and sex were balanced among the litters. Three treatments were randomly assigned to the litters within each room (six litters/treatment), which were also arranged in a factorial array. The treatments (factors) included copper supplementation from Cu sulfate (CuSO_4_): (i) a low-copper (LC, 6 mg kg^−1^) diet containing no supplemental Cu; (ii) a control (CON, 20 mg kg^−1^) diet; or (iii) a high-copper (HC, 300 mg kg^−1^) diet. The prefeeding began in suckling piglets at 7 to 14 days of age, and the piglets were trained to feed every day. With the increase in feed intake, the amount of nursing was reduced. The corn/soybean-based diets were supplied throughout the experiment, which included the prefeeding period, conformed to the nutrient requirements of the National Research Council (Southern et al. 2012b) (Table 1). The piglets were allowed free access to feed and water during the 26-day (14–40 days of age) animal trial. Prior to weaning, three litters were chosen within each treatment, and blood and feces samples were collected from four piglets within each litter (two male and two female). Blood samples were obtained using glass tubes containing no anticoagulant, allowed to clot at room temperature and stored at 4 °C before harvest of serum by centrifugation (15 min at 3500 rpm). The serum and fecal samples were stored at − 80 °C for subsequent analyses.

**Table 1:** Composition of the experimental diets (mean ± SD)

Items	Cu supplementation, mg kg^−1^ diet		
	LC (6)	CON (20)	HC (300)
Ingredient (%)			
Corn		69.00	
Soybean meal		14.00	
Extruded soybeans		5.00	
Self-made premix^a^		12.00	
Nutrition level (%)^b^			
Dry matter		87.24	
Crude protein		17.86	
Crude fat		1.98	
Crude fiber		4.15	
Ash		4.76	
Measured Cu value (μg g^−1^)	5.90 ± 1.04	23.14 ± 3.59	295.31 ± 14.80

^a^Ingredients: fish meal, choline chloride, vitamin, mineral elements, l-lysine hydrochloride, calcium hydro phosphate, stone powder, sodium chloride, enzyme preparation, flavoring agent, and sweetening agent. No antibiotics were added

^b^Measured values

### Copper measurement

The copper content in the fecal samples from suckling piglets were measured by inductively coupled plasma optical emission spectrometry (ICP-OES) (PerkinElmer, Waltham, USA). The feces (500 μg) were dried and placed in a tube with 10 mL of a mixture of nitric acid (chemically pure) and perchloric acid (chemically pure) (3:1 *v*/*v*). After digestion overnight, tubes were heated from 100 to 240 °C for approximately 3 h, and then the digests were brought to a constant volume with double-distilled deionized water (Zhang et al. 2017a).

### DNA extraction, 16S rRNA gene amplicon pyrosequencing, and sequence analysis

The methods in this section are similar to those of our previous studies (Zhang et al. 2017a), in which the DNA was extracted from the piglet fecal samples with a QIAamp Fast DNA Stool Mini Kit (QIAGEN, Duesseldorf, Germany) and quantified with a NanoDrop spectrophotometer (NanoDrop 2000, NanoDrop Technologies, Waltham, USA).

The polymerase chain reaction (PCR) (95 °C for 5 min, followed by 27 cycles of 95 °C for 30 s, 55 °C for 30 s, and 72 °C for 45 s and a final extension at 72 °C for 10 min) was used to amplified the V4–V5 region of the 16S rRNA gene; the primers were 341F (5′-CCTAYGGGRBGCASCAG-3′) and 806R (5′-GGACTACNNGGGTATCTAAT-3′). The AxyPrep DNA Gel Extraction Kit (Axygen Biosciences, Tewksbury, USA) and the QuantiFluor-ST kit (Promega, Madison, USA) were used to purify and quantify all PCR products. The purified amplicons were sequenced on an Illumina MiSeq platform by Biozeron Biotechnology (Shanghai, China). The raw reads were deposited into the National Center for Biotechnology Information (NCBI) Sequence Read Archive database (Accession Number SRP155696).

Raw fastq files were demultiplexed, quality-filtered using Quantitative Insights Into Microbial Ecology (QIIME, version 1.17, http://qiime.org/scripts/assign_taxonomy.html) with the following criteria: the 250-bp reads were truncated at any site receiving an average quality score < 20 over a 10-bp sliding window, discarding the truncated reads that were shorter than 50 bp; exact barcode matching, 2-nucleotide mismatch in primer matching, reads containing ambiguous characters were removed; only sequences that overlapped by more than 10 bp were assembled according to their overlap sequence. Reads which could not be assembled were discarded. Operational taxonomic units (OTUs) were clustered with 97% similarity cutoff using UPARSE (version 7.1, http://drive5.com/uparse/) and chimeric sequences were identified and removed using UCHIME (Sun et al. 2016). The phylogenetic affiliation of each 16S rRNA gene sequence was analyzed by RDP Classifier (http://rdp.cme.msu.edu/) against the SILVA (SSU123, https://www.arb-silva.de) 16S rRNA database using a confidence threshold of 70% (Amato et al. 2013).

### Serum biochemical parameters analysis

The serum inflammatory cytokines, oxidative and antioxidative enzymes, and indices of hepatic and renal function were measured with detection kits produced by Nanjing Jiancheng Bioengineering Institute (Nanjing, Jiangsu province, China); all examinations were performed according to the manufacturer’s instructions.

### Sample preparation for gas chromatography-mass spectrometer analysis

Fecal samples (100 mg) were transferred into 5-mL centrifuge tubes; 500 μL of ddH_2_O (4 °C) was added, and the tubes were vortexed for 60 s. Next, 1 mL of methanol (precooled at − 20 °C) and 60 μL of heptadecanoic acid (0.2 mg mL^−1^ stock in methanol), as an internal quantitative standard, were added and vortexed for 30 s. The tubes were then placed into an ultrasound machine at 25 °C for 10 min, incubated on ice for 30 min, and centrifuged for 10 min at 12,000 rpm (4 °C), after which 1.2 mL of the supernatant was transferred into a new centrifuge tube. Samples were blow-dried by vacuum concentration, and then 60 μL of 15 mg mL^−1^ methoxyamine pyridine solution was added. The sample was vortexed for 30 s and reacted for 120 min at 37 °C. Finally, 60 μL of bis(trimethylsilyl)trifluoroacetamide reagent (containing 1% trimethyl chlorosilane) was added to the mixture, reacted for 90 min at 37 °C, and centrifuged at 12,000 rpm 4 °C for 10 min, and the supernatant was transferred into a bottle for inspection; 20 μL of each sample extract was used for quality control (QC), and the remaining sample material was used for gas chromatography-mass spectrometer (GC-MS) test detection.

### GC-MS analysis of fecal metabolite profiles

The derivatized sample (1.0 μL) was injected in split mode into an Agilent 7890A (Agilent, CA, USA) system in a 20:1 split ratio using an autosampler. Gas chromatography was performed on an HP-5MS capillary column (Folsom, CA, USA) to separate the derivatives at a constant flow of 1 mL min^−1^ helium. The injection temperature was 280 °C, the interface was set to 150 °C, and the ion source was adjusted to 230 °C. The temperature-rise programs were followed by an initial temperature of 60 °C for 2 min, 10 °C min^−1^ up to 300 °C, and a steady 300 °C for 5 min. Mass spectrometry (Agilent 5975C, Agilent, CA, USA) was determined in the full-scan method with a 35 to 750 (*m z*^−1^) range.

### GC-MS data acquisition and processing

Following raw data collection, compounds were identified by comparing the mass spectra and retention indices of all detected compounds with their reference standards and database in the National Institute of Standards and Technology (NIST, https://www.nist.gov/srd) database and NEW Wiley 9 mass spectra library database (Sun et al. 2016). The SIMCA-p software (version 13.0, Umetrics, Umea, Sweden) was used to conduct multivariate statistical analysis. The acquired GC-MS data were processed with partial least squares-discriminant analysis (PLS-DA) (Sun et al. 2016). The metabolites with variable important projection (VIP) values > 1.0 and one-way analysis of variance (ANOVA) *P* values < 0.05 were considered as different metabolites among the three dietary groups. The MetaboAnalyst (v4.0, http://www.metaboanalyst.ca/faces/ModuleView.xhtml) online tool was used to process the metabolic pathways and metabolite set enrichment analysis (Xia et al. 2009).

### Statistical analysis

The relative abundance of microbial communities in feces and data that did not follow a normal distribution were processed using the nonparametric Kruskal-Wallis test. The correlation analysis was performed by the Spearman or Pearson correlation tests. Significant differences were considered as *P* < 0.05. The statistical analyses were conducted using SPSS Statistics (Version 22, https://www.ibm.com/analytics/spss-statistics-software) (Zhang et al. 2017a).

## Results

### Dietary copper level affects the copper content in feces of suckling piglets

As shown in Table 2, the fecal copper content in the HC group was significantly higher than that in the LC and CON groups (*P* < 0.05); there were no significant differences in fecal copper content between the LC and CON group (*P* > 0.05; Table 2).

**Table 2:** Copper content in the feces of suckling piglets (mean ± SD)

Items	Cu supplementation, mg kg^−1^ diet			SEM	*P* value
	LC (6)	CON (20)	HC (300)		
Fecal copper content (μg g^−1^)	195.01 ± 34.55^b^	275.62 ± 54.65^b^	1239.37 ± 602.12^a^	104.42	< 0.05

Values within a row without a common superscript letter are significantly different (*P* < 0.05)

### Effect of dietary copper level on the composition of fecal microbiota

At a cutoff level of 3%, no effect on the number of reads or OTUs was observed among the groups. The richness estimator Chao1 tended to be decreased in the CON group (*P* = 0.07); there were no significant differences in the richness estimator ACE or the Shannon and Simpson diversity indices among the groups (*P* > 0.05; Table 3).

**Table 3:** Effect of dietary copper levels on the richness and diversity of fecal microbiota in suckling piglets (mean ± SD)

Items	Cu supplementation, mg kg^−1^ diet			SEM	*P* value
	LC (6)	CON (20)	HC (300)		
Reads	37,303.73 ± 3732.58	36,596.09 ± 4675.63	35,454.42 ± 4924.50	758.09	0.61
OTUs	628.00 ± 75.80	580.27 ± 33.92	627.08 ± 60.39	10.58	0.11
ACE	728.43 ± 84.56	681.21 ± 44.48	734.80 ± 58.55	11.49	0.12
Chao1	742.31 ± 79.03	684.11 ± 55.32	744.85 ± 63.08	12.06	0.07
Shannon	4.70 ± 0.25	4.59 ± 0.12	4.64 ± 0.28	0.04	0.51
Simpson	0.022 ± 0.008	0.023 ± 0.005	0.025 ± 0.010	0.001	0.71

**Fig. 1:**
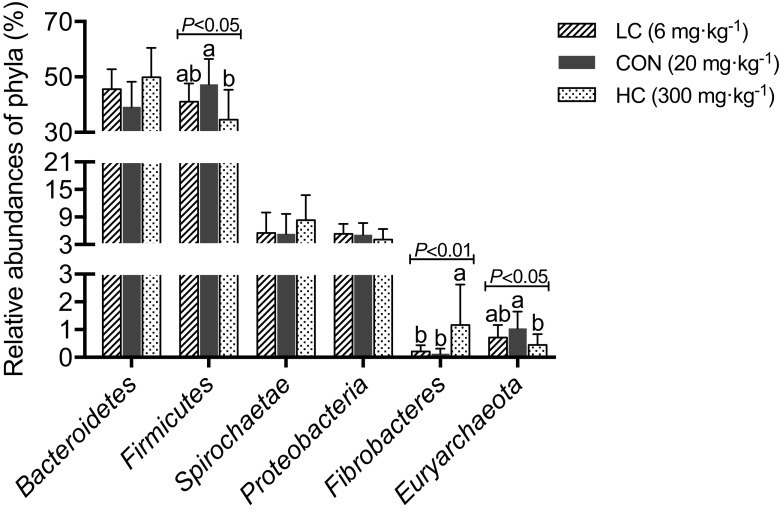
Effect of dietary copper level on the relative abundance of phyla in suckling piglets (relative abundance > 1%)

At the phylum level, *Bacteroidetes* and *Firmicutes* were the predominant phyla in the fecal microbiota of piglets, with a total abundance > 90%, followed by the phyla *Spirochaetes*, *Proteobacteria*, *Fibrobacteres*, and *Euryarchaeota* (Supplemental Fig. S1). The relative abundance of *Firmicutes* and *Euryarchaeota* was higher in the CON group than in the HC group, while the relative abundance of *Fibrobacteres* was higher in the HC group compared to the other groups (*P* < 0.05; Fig. 1).

**Fig. 2:**
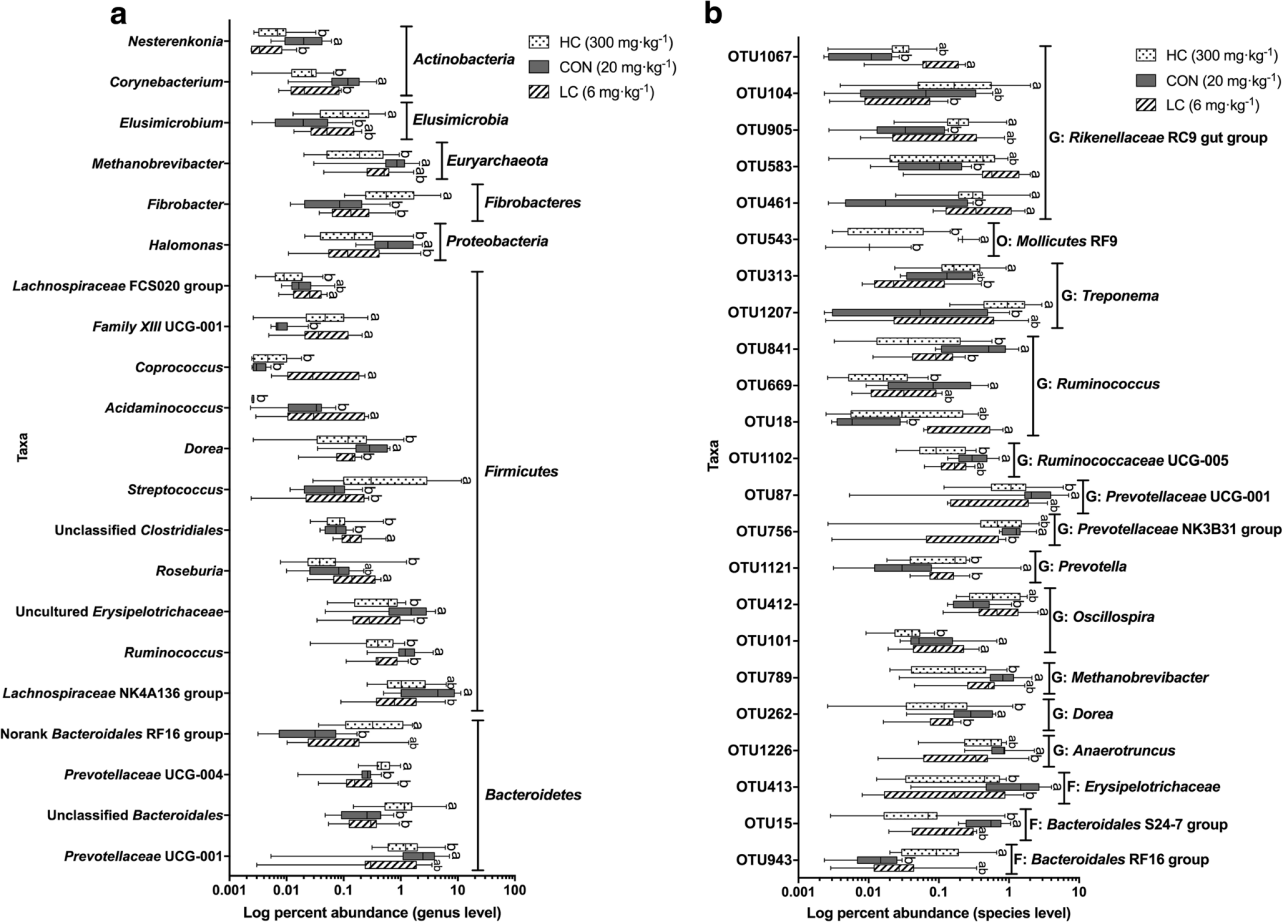
Effect of dietary copper level on the relative abundances of fecal microbiota genera and OTUs in suckling piglets. **a** Genus level. **b** OTU level

At the genus level, the relative abundance levels of *Ruminococcus*, *Dorea*, and *Corynebacterium* were higher in the CON group and of *Prevotellaceae* UCG-004 in the HC group than in the other groups (*P* < 0.05). The relative abundance levels of *Prevotellaceae* UCG-001, *Lachnospiraceae* NK4A136 group, and *Nesterenkonia* were lower, while *Coprococcus* abundance was increased in the LC group compared to the CON group (*P* < 0.05); the abundance levels of *Streptococcus* and *Fibrobacter* were lower and the abundance levels of *Halomonas* and *Methanobrevibacter* were higher in the HC group than in the CON group; and the abundance levels of *Acidaminococcus* and *Lachnospiraceae* FCS020 group were lower in the HC group than in the LC group (*P* < 0.05; Fig. 2a).

At the OTU level, the abundance levels of *Oscillospira*-, *Rikenellaceae*-, and *Ruminococcus*-related OTUs were lower, while *Dorea*-, *Prevotella*-, *Ruminococcus*-, and *Anaerotruncus*-related OTUs were higher in the CON group than in the LC group (*P* < 0.05); the abundance levels of *Treponema*- and *Rikenellaceae*-related OTUs were higher, while those of *Methanobrevibacter*-, *Ruminococcus*-, *Dorea*-, *Prevotella*-, and *Oscillibacter*-related OTUs were lower in the HC group than in the CON group (*P* < 0.05); and the abundance levels of *Treponema*- and *Rikenellaceae*-related OTUs were higher in the HC group than in the LC group (*P* < 0.05; Fig. 2b).

### Dietary copper levels affect the biochemical parameters in the serum of suckling piglets

**Fig. 3:**
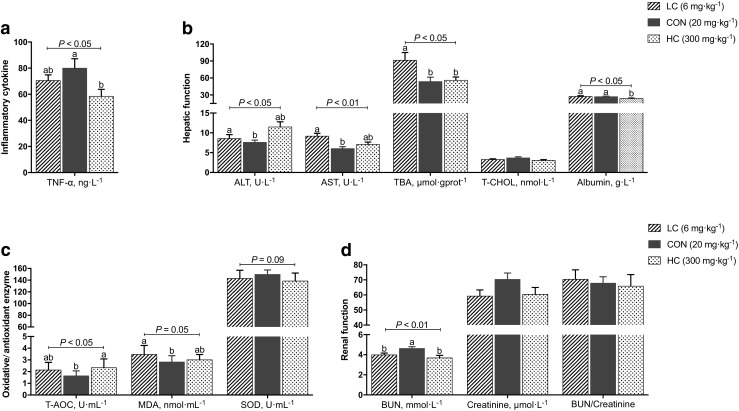
Effect of dietary copper on serum biochemical parameters in suckling piglets. **a** Inflammatory cytokine. **b** Hepatic function. **c** Oxidative/antioxidative enzyme. **d** Renal function. TNF-α, tumor necrosis factor-α; ALT, alanine aminotransferase; AST, aspartate transaminase; TBA, total bile acid; T-CHOL, total cholesterol; T-AOC, total antioxidant capacity; MDA, malondialdehyde; SOD, superoxide dismutase; BUN, blood urea nitrogen

Compared with the CON group, TNF-α (*P* < 0.05) was decreased and the total antioxidant capacity (T-AOC) (*P* < 0.05) was increased in the HC group. Meanwhile, malondialdehyde (MDA) (*P* = 0.05), alanine aminotransferase (ALT) (*P* < 0.05), aspartate transaminase (AST) (*P* < 0.01), and total bile acid (TBA) (*P* < 0.05) were increased in the LC group; serum albumin (*P* < 0.05) was decreased in the HC group compared with the LC and CON groups; the serum blood urea nitrogen (BUN) (*P* < 0.01) was increased, and the superoxide dismutase (SOD) (*P* = 0.09) tended to be increased in the CON group compared with the LC and HC groups (Fig. 3).

### Correlations between fecal microbiota and serum biochemical parameters

**Fig. 4:**
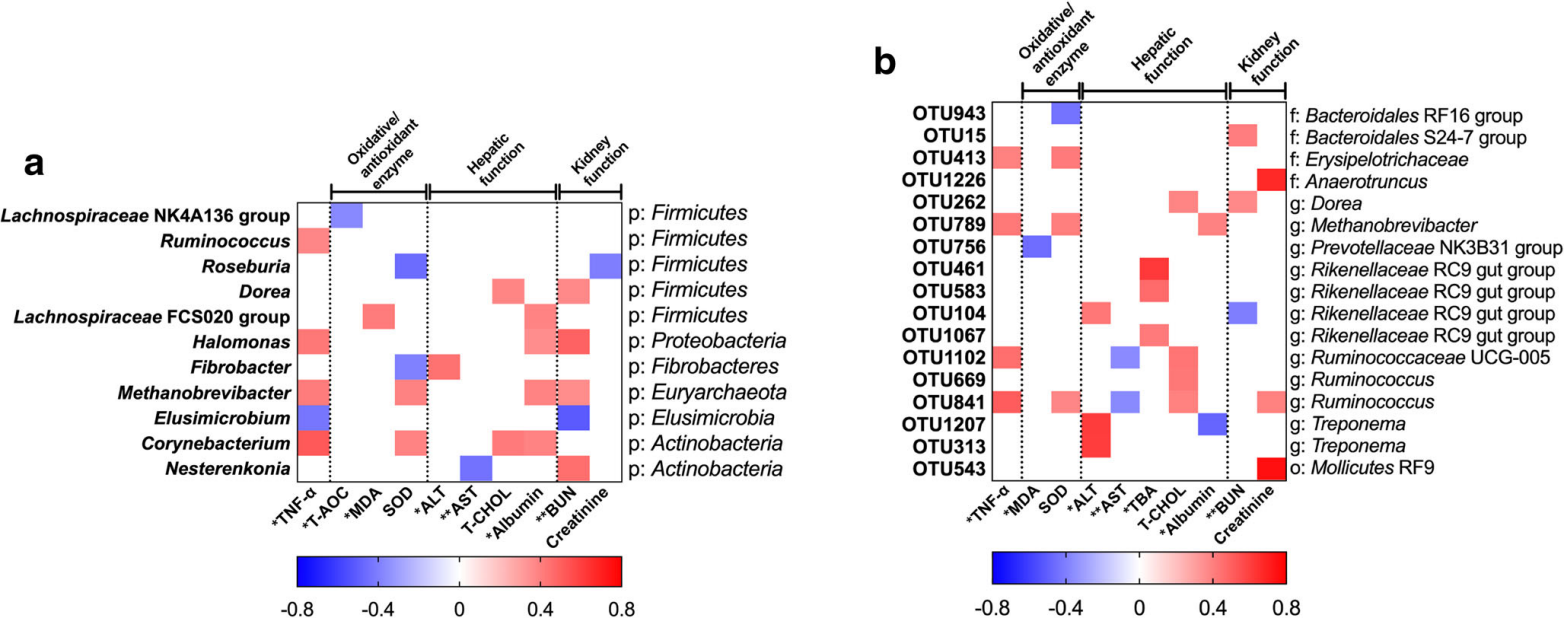
Correlation analysis of microbiota (genus and OTU levels) and inflammatory cytokine, oxidation reduction, and hepatorenal function. **a** Genus level. **b** OTU level. The color is according to the Pearson coefficient distribution: red represents a positive correlation (*P* < 0.05), blue represents a negative correlation (*P* < 0.05), and white shows that the correlation was not significant (*P* > 0.05). *Significantly affected by dietary copper level (*P* < 0.05); **Significantly affected by dietary copper level (*P* < 0.01); p, phylum; o, order; f, family; g, genus

At the genus level, the abundance levels of *Lachnospiraceae* (NK4A136 and FCS020 groups) were positively correlated with MDA and albumin and negatively correlated with T-AOC. The abundance of *Ruminococcus* was positively correlated with TNF-α. The abundance levels of *Halomonas* and *Methanobrevibacter* were positively correlated with TNF-α, albumin, and BUN. The abundance of *Corynebacterium* was positively correlated with TNF-α and albumin (Fig. 4a).

At the OTU level, the abundance levels of OTU413 (*Erysipelotrichaceae*), OTU789 (*Methanobrevibacter*), and OTU841 (*Ruminococcus*) were positively correlated with TNF-α and SOD (*P* < 0.05); the abundance level of OTU756 (*Prevotellaceae* NK3B31 group) was negatively correlated with MDA (*P* < 0.05); the abundance levels of OTU104 (*Rikenellaceae* RC9 gut group), OTU1207 (*Treponema*), and OTU313 (*Treponema*) were positively correlated with ALT (*P* < 0.05); the abundance levels of OTU1102 (*Ruminococcaceae* UCG-005) and OTU841 (*Ruminococcus*) were negatively correlated with AST (*P* < 0.05); the abundance levels of *Rikenellaceae* RC9 gut group (OTU461, OTU583, and OTU1067) were positively correlated with TBA (*P* < 0.05); and the abundance levels of OTU15 (*Bacteroidales* S24-7 group) and OTU262 (*Dorea*) were positively correlated with BUN (*P* < 0.05) (Fig. 4b).

### Effect of dietary copper level on fecal metabolite profiles

Overall, a total of 205 nontargeted peaks were detected, and 91 compounds were annotated based on a comparison with reference compounds in available libraries or authentic reference standards. These metabolites included amino acids, carbohydrates, fatty acids, amines, polyols, organic acids, and nucleotides involved in multiple biochemical processes in suckling piglets. The key compounds responsible for differentiation were identified using the multivariate analysis method of PLS-DA (Supplemental Fig. S2).

**Fig. 5:**
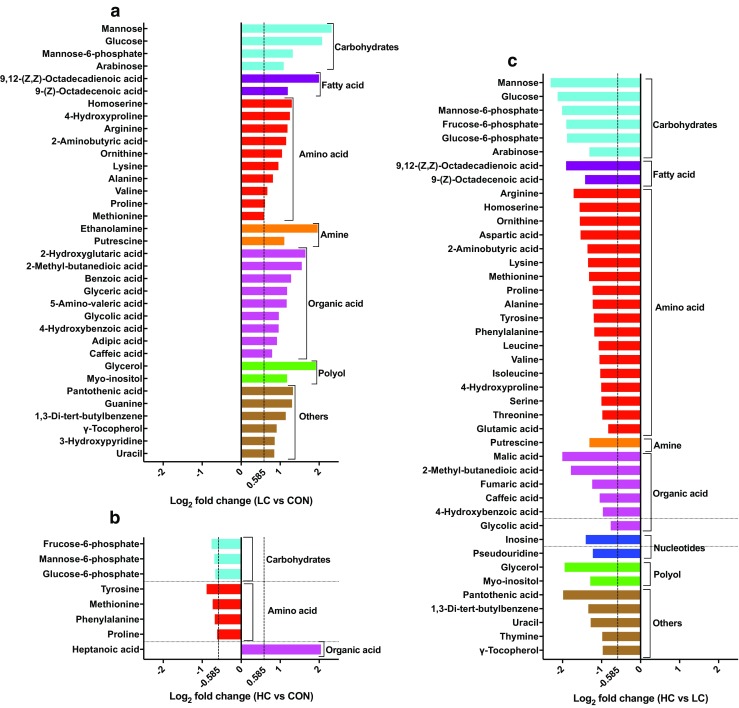
Fold change of significant fecal metabolites. Significant metabolites accountable for class discrimination with VIP > 1 and *P* < 0.05 are listed. The fold change was calculated using the normalized relative abundance of metabolites between each group: **a** LC vs. CON; **b** HC vs. CON; and **c** HC vs. LC. The threshold was 0.585; enrichment > 0.585, reduction ≤ -0.585

**Fig. 6:**
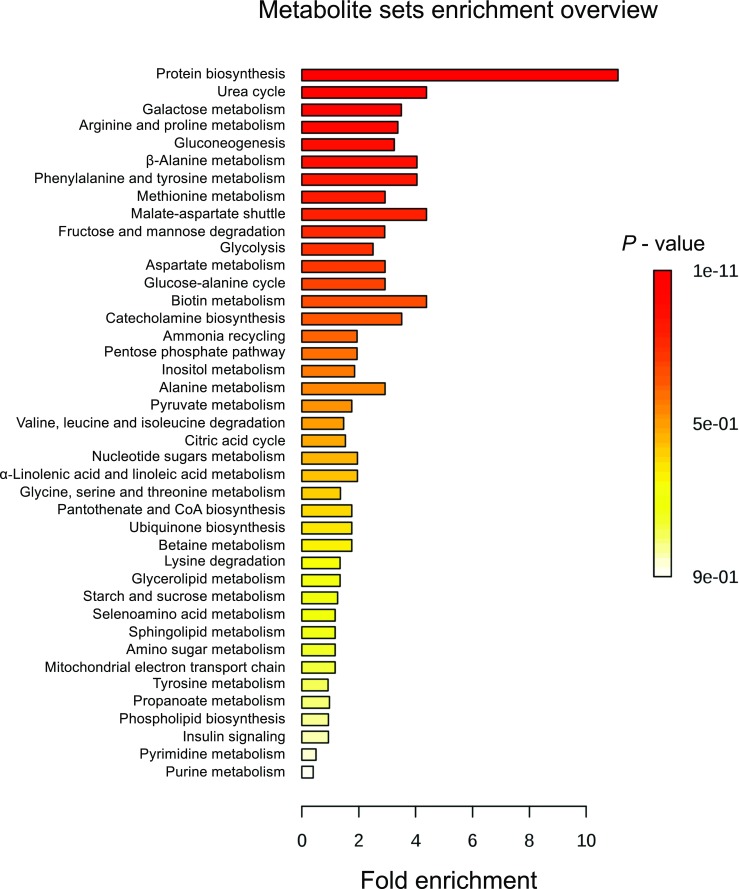
Metabolic pathway enrichment analysis. Overview of metabolites that were enriched in suckling piglets fed different levels of dietary copper

Criteria including VIP > 1, *P* < 0.05, and one-way ANOVA *P* < 0.05 were used to identify the compounds responsible for the difference between two and three dietary groups. A total of 47 significant fecal metabolites were identified for the three dietary groups (Supplemental Table S1). Compared with the CON group, 35 compounds were enriched in the LC group (Fig. 5a) and seven compounds were reduced and one compound was enriched in the HC group (Fig. 5b). Compared with the LC group, 42 compounds were reduced in the HC group (Fig. 5c). Further metabolic pathway enrichment analysis showed that dietary copper level had significant effects on protein biosynthesis, the urea cycle, galactose metabolism, gluconeogenesis, and amino acid metabolism (including the metabolism of arginine, proline, β-alanine, phenylalanine, tyrosine, and methionine; Fig. 6).

### Correlations between fecal microbiota and significant metabolites

**Fig. 7:**
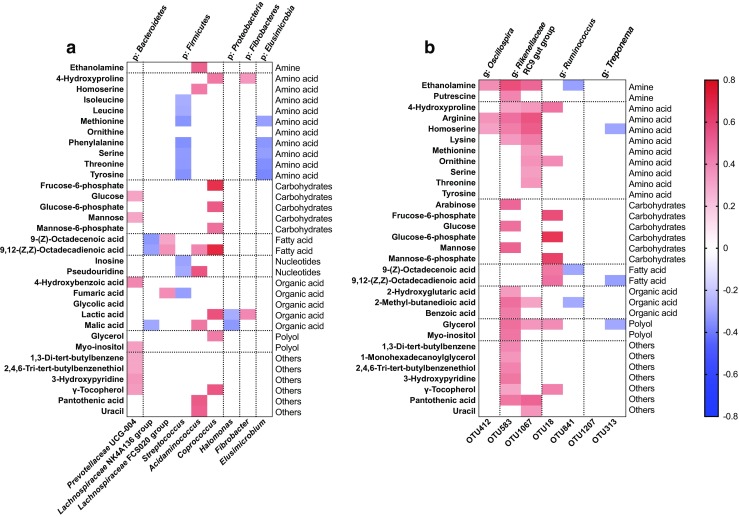
Correlations between fecal microbiota and significant metabolites. Statistically significant correlations are presented, colored according to the Pearson coefficient distribution: red represents a positive correlation (*P* < 0.05), blue represents a negative correlation (*P* < 0.05), and white indicates that the correlation was not significant (*P* > 0.05); p, phylum; g, genus

Fecal metabolites, fecal microbial genera, and OTUs with significant differences among the three groups were subjected to Pearson’s correlation analysis. At the genus level, the relative abundance of *Prevotellaceae* UCG-004 was positively correlated with carbohydrates (glucose and mannose), organic acids (4-hydroxybenzoic acid), or polyols (myo-inositol) (*P* < 0.05); the relative abundance of *Streptococcus* was negatively correlated with amino acids (isoleucine, leucine, methionine, phenylalanine, serine, threonine, and tyrosine), nucleotides (inosine and pseudouridine), and organic acids (fumaric acid) (*P* < 0.05); and the relative abundance of *Acidaminococcus* was positively correlated with amines (ethanolamine), amino acids (homoserine), 9,12-(z,z)-octadecadienoic acid, nucleotides (pseudouridine), and organic acids (malic acid) (*P* < 0.05; Fig. 7a).

At the OTU level, the relative abundance of OTU583 was positively correlated with amines (ethanolamine and putrescine), amino acids (4-hydroxyproline, arginine, homoserine, and lysine), carbohydrates (arabinose, glucose, and mannose), organic acids (2-hydroxyglutaric acid, 2-methyl-butanedioic acid, and benzoic acid), and polyols (glycerol and myo-inositol) (*P* < 0.05); the relative abundance of OTU1067 was positively correlated with amines (ethanolamine), amino acids (4-hydroxyproline, arginine, homoserine, lysine, methionine, ornithine, serine, and tyrosine), organic acids (2-methyl-butanedioic acid), and polyols (glycerol) (*P* < 0.05); and the relative abundance of OTU18 was positively correlated with amino acids (4-hydroxyproline and ornithine), carbohydrates (fructose-6-phosphate, glucose-6-phosphate, and mannose-6-phosphate), and polyols (glycerol) (*P* < 0.05; Fig. 7b).

**Fig. 8:**
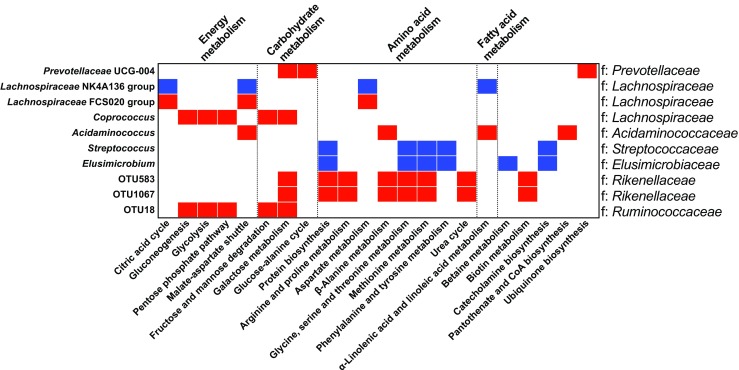
Correlations between microbiota and enriched fecal metabolite pathways. Red represents a positive correlation (*P* < 0.05), blue represents a negative correlation (*P* < 0.05), and white indicates that the correlation was not significant (*P* > 0.05); f, family

The significant fecal metabolites correlated with microbial genera and OTUs were used for further metabolic pathway enrichment analysis. The relative abundance levels of *Coprococcus* (family *Lachnospiraceae*) and OTU18 (family *Ruminococcaceae*) were positively correlated with energy metabolism pathways (gluconeogenesis, glycolysis, and pentose phosphate pathway); the abundance of *Streptococcus* was negatively correlated with amino acid metabolism pathways (protein biosynthesis, glycine, serine, threonine, methionine, phenylalanine, and tyrosine metabolism); and the abundance levels of OTU583 and OTU1067 (family *Rikenellaceae*) were positively correlated with amino acid metabolism pathways (protein biosynthesis, β-alanine, glycine, serine, threonine, and methionine metabolism; Fig. 8).

## Discussion

It has been estimated that between 500 and 1000 bacterial species populate in the gastrointestinal (GI) tract, accounting for a total of approximately 10^14^ bacteria (Sonnenburg et al. 2004). In this study, most of the dietary copper in the HC group was not absorbed and was instead accumulated in the feces of suckling piglets, which is consistent with our previous studies in rats (Zhang et al. 2017a). Whether high levels of copper in the environment in which bacteria survive affect microbial communities in piglets requires further discussion.

The richness (ACE and Chao indices) and diversity (Shannon and Simpson indices) are important features of microbial communities (Chao et al. 1993; Shen et al. 2003). In our study, the richness and diversity indices were not affected by dietary copper level, in contrast to a previous study reporting that high dietary copper levels (100 to 200 mg kg^−1^) from CuSO_4_ significantly affect microbial species (diversity) in ileac (Namkung et al. 2006), cecal (Hojberg et al. 2005; Mei et al. 2010), and colonic (Namkung et al. 2006) chyme of piglets. In addition, microbial diversity was decreased in a 250 mg kg^−1^ Cu diet compared with a diet containing no copper supplementation (Namkung et al. 2006). A possible reason for this difference is that the method used to investigate the microbial flora is inconsistent. In the GI ecosystem, most bacterial species have yet to be cultured. Because of the high costs and low throughput, studies on the diversity of microflora in the GI tract have been limited, even by using PCR, cloning, and sequencing as culture-independent methods. However, the recent application of 16S rRNA gene sequence analysis and the development of pyrosequencing technology have enabled a more comprehensive exploration of microbial communities in the GI tract and their diversity (Chakravorty et al. 2007; Huber et al. 2007; Sogin et al. 2006); therefore, the approach of analyzing microbial communities based on 16S rRNA pyrosequencing is deemed more suitable (Dewar et al. 2013).

In our study, *Firmicutes* and *Bacteroidetes* were the most dominant phyla, similar to the findings of previous studies (Li et al. 2017; Qin et al. 2010); low-level copper dietary supplementation (36–47 mg kg^−1^) from montmorillonite and high-level (200 mg kg^−1^) copper supplementation from CuSO_4_ or CuO decreased the number of *Clostridia* in the cecum and colon of pigs (Hu et al. 2004; Song et al. 2013; Xia et al. 2005). Our 16S rRNA gene pyrosequencing analysis demonstrated that dietary copper levels affected the relative abundance of several *Clostridia* genera, including *Coprococcus*, *Dorea*, *Roseburia*, and *Ruminococcus*. However, changes in these genera were inconsistent; the abundance levels of butyrate-producing bacteria, such as *Coprococcus*, *Roseburia* (Duck et al. 2007; Klaring et al. 2015), and *Acidaminococcus* (Braune et al. 1999; Jumas-Bilak et al. 2007), were decreased in the HC group, suggesting that high levels of dietary copper (300 mg kg^−1^) might inhibit the growth of beneficial bacteria in the gut. The decreased abundance of butyrate-producing bacteria in the hindgut of piglets led to an increased pH, while short-chain fatty acids (SCFA) reduced and weakened nutrient (such as dietary fiber) competition with other bacteria, as indicated by the increased abundance levels of the genera *Streptococcus* and *Fibrobacter* in the HC group. Most genus *Streptococcus* species are urea-decomposing bacteria, which could degrade the dietary nitrogen compounds into ammonia via the produced urease (Varel et al. 1987). The increased abundance of *Streptococcus* in the HC group might accelerate this degradation and elevate ammonia levels, causing toxic effects in the hindgut of piglets.

As we know, dietary different copper levels may affect the physiological parameters in the body, such as blood. To investigate the relationship between physiological parameters and fecal microbial communities in suckling piglets, the serum biochemical parameters were further analyzed. A decrease in serum TNF-α concentration in the HC group was observed in our study, suggesting that without antibiotic use, high levels of copper may have a potential role in decreasing the inflammatory response. Considering that SOD is a group of metalloenzymes that protect cells from superoxide radicals by degrading superoxide radicals into hydrogen peroxide (Hao et al. 2015), in the present study, the serum SOD level increased and MDA was decreased in the CON group compared with the LC and HC groups remarkably, suggesting that 20 mg kg^−1^ Cu in the diet could effectively improve the antioxidant ability and protect tissues from oxidative damage. Because pigs fed a 6 mg kg^−1^ Cu diet suffered increased oxidative stress, which induced possible damage in the liver, a hepatocellular injury usually results in the elevation of serum ALT and AST levels (Chang et al. 2011; Masubuchi et al. 2016), similarly, our results observed that serum ALT and AST were increased in the LC and HC groups compared with the CON group in suckling piglets. In our study, serum bile acids in the LC group were increased compared with the CON group. The measurement of changes in serum bile acids was considered a valuable biomarker, which is widely used for the diagnosis of hepatic disease (Fanali et al. 2012; Iwamura 1982; Masubuchi et al. 2016). Taking these results together, a dietary dose of 6 mg kg^−1^ Cu did not maintain normal liver function, and 300 mg kg^−1^ Cu might both have the potential risk of impaired liver function in suckling piglets. In the current study, dietary copper levels did not affect renal function due to the result of BUN/creatinine, elevation of which is considered a serum marker for identifying acute kidney injury (AKI) (Takaya et al. 2015); the body tissue was able to excrete any minerals above those needed by the body and did not store them in the kidney (Gowanlock et al. 2015).

Then, the correlation between fecal microbiota significantly affected by dietary copper level and serum biochemical parameters from suckling piglets were further analyzed in our study, helping us to understand the relationship between changes in gut microbiota and host health. In our study, the abundance of *Lachnospiraceae* was affected by dietary copper levels. These bacteria are the most active microbial components in the gut of healthy adults (Peris-Bondia et al. 2011), preventing the production of inflammatory cytokines and pathogen-induced intestinal dysfunction by fermenting carbohydrates into SCFA (Duncan et al. 2007; Reiff and Kelly 2010). The abundance of *Lachnospiraceae* was negatively correlated with T-AOC and positively correlated with MDA in serum, suggesting that these bacteria may cause changes in the redox balance of suckling piglets (Li et al. 2016). The abundance levels of *Ruminococcus*, *Methanobrevibacter*, and *Corynebacterium* were positively correlated with TNF-α in serum, which increased in the CON group, suggesting that altering these bacterial communities may influence the inflammatory response in piglets, consistent with our previous studies in rats (Zhang et al. 2017a). Meanwhile, correlation analysis shows that many bacteria were correlated with redox state and hepatic and renal function of piglets, and the abundance levels of these bacteria were affected by dietary copper level, which also suggested that the alteration of microbial communities may influence the serum biochemical parameters, further affecting the health of piglets.

Fecal metabolites reflect the final status of animal digestion, absorption, and metabolism of feed nutrients. The essential amino acids leucine, phenylalanine, and methionine and the conditionally essential amino acids proline and tyrosine were higher in the LC group than in the HC group (Supplemental Fig. S3), suggesting that the capacity or dietary protein digestion, absorption, and metabolism were lower in suckling piglets those fed with 6 mg kg^−1^ Cu diet. A growing number of recent studies have shown that proline and leucine are involved in body protein synthesis (Beitz 2004; Columbus et al. 2015; Hernandez-Garcia et al. 2016; Manjarin et al. 2016; Soumeh et al. 2015; Zhang et al. 2017b) and animal growth (Festa and Thiele 2011; Kim et al. 2008; Mao et al. 2015) and that the metabolites 4-hydroxyproline, alanine, tyrosine, and methionine are involved with the tricarboxylic acid cycle (TCA) cycle (Beitz 2004), which provides the energy for organism life activities and is also connected with carbohydrate, fat, and protein metabolism. Taken together, these findings suggest that feed with 6 mg kg^−1^ Cu supplementation has an adverse effect on the growth of piglets and may affect the health of piglets through the TCA cycle.

In pigs, dietary carbohydrates are mainly digested in the small intestine and absorbed as monosaccharides. Some carbohydrates are fermented in the form of SCFA following fermentation by intestinal microbes. Carbohydrates that have not been absorbed and degraded by the host are excreted in the feces (Southern et al. 2012a). In this study, arabinose, mannose, fructose-6-phosphate, mannose-6-phosphate, and glucose-6-phosphate levels were significantly higher in the LC than in the CON or HC group (Supplemental Fig. S3). These results suggest that dietary 6 mg kg^−1^ copper decreased monosaccharide absorption in piglets.

Diarrhea is a major health problem for piglets during growth. Previously, we found that the incidence of diarrhea was higher in piglets supplemented with 6 mg kg^−1^ Cu than in the 20 and 300 mg kg^−1^ groups, which further confirmed the significantly upregulated levels of amines (putrescine) in the LC (6 mg kg^−1^) group (Supplemental Fig. S3). Amines are produced via the decarboxylation of amino acids by intestine microbiota and are rapidly absorbed in the colon. The enzyme diamine oxidase in the intestinal mucosa can catalyze the oxidation of histamines, putrescines, and other diamines to aldehydes for detoxification (Festa and Thiele 2011; Kim et al. 2008) via the liver or urine discharge (Htoo et al. 2007). Excessively high dietary protein levels or decreased digestion and absorption in the small intestine can increase the fermentation of amino acids by large intestine microbiota, leading to increased levels of putrescine and other amines and subsequently resulting in diarrhea in piglets (Gaskins 2001). In addition, copper is a cofactor of diamine oxidase; therefore, dietary copper levels may affect the level of amines (putrescine) in the gut and the incidence of diarrhea in piglets. Lactic acid and succinic acid are intermediate products of bacterial synthesis of propionic acid and butyric acid; the levels of these acids are low or undetectable in the gut when the microbiota structure remains stable. In the absence of lactic acid– and succinic acid–utilizing bacteria, intestinal epithelial cells slowly absorb and accumulate lactic acid and succinic acid, resulting in an increase in the osmotic pressure in the large intestine lumen, leading to increased intestinal mucosal water secretion and subsequently diarrhea. In this study, the levels of lactate and succinic acid were increased in fecal samples from LC (6 mg kg^−1^) piglets, which may be another reason for the higher diarrhea rate. In addition, the levels of organic acids were increased in the feces of piglets in the LC (6 mg kg^−1^) group (Supplemental Fig. S3), and this excessive level of organic acids decreased the pH in the hindgut; previous studies have reported that reduced pH in the intestine can prevent the colonization of pathogenic bacteria; however, low pH (pH = 5) can lead to impaired normal intestine function in rats and pigs and the inability to maintain water absorption in the gut lumen (Argenzio and Meuten 1991; Tsukahara and Ushida 2001).

Piglet feces metabolites can be divided into two groups: the body and the gut microbiota. The relative abundance of *Prevotellaceae* UCG-004 was increased in the HC (300 mg kg^−1^) group, and *Prevotella* was the major fiber-degrading bacterium in the porcine intestine. Metabolomic analysis showed that the digestibility of carbohydrates in piglets from the HC (300 mg kg^−1^) group increased, and the correlation and enrichment analysis demonstrated that the abundance of *Prevotellaceae* UCG-004 was significantly positively correlated with galactose metabolism and the glucose-alanine cycle (Fig. 8), suggesting that dietary copper levels may affect the digestion and utilization of carbohydrates by influencing the relative abundance of bacteria associated with carbohydrate metabolism in the suckling piglet intestine. The family *Lachnospiraceae* genera *Coprococcus* and *Acidaminococcus* produce butyrate via the pyruvate pathway; their relative abundance levels were significantly positively correlated with carbohydrate metabolic pathways (citrate cycle, malate shuttle, gluconeogenesis, pentose phosphate pathway, and glycolysis; Fig. 8). The abundance of OTU18 (*Ruminococcus*) was positively correlated with carbohydrate metabolism pathways (glycolysis, gluconeogenesis, pentose phosphate pathway, fructose, mannose, and galactose metabolism), and the abundance levels of OTU583 and OTU1067 (*Rikenellaceae* RC9 gut group) were positively correlated with small molecule containing N metabolism pathways (protein biosynthesis, urea cycle, methionine, glycine, serine, threonine, arginine, and valine metabolism; Fig. 8). The abundance levels of these bacteria were increased in the LC (6 mg kg^−1^) group, suggesting that dietary copper level may affect the metabolism of carbohydrates and small N-containing molecules in piglets by altering the abundance of these bacteria to some extent. Interestingly, dietary copper (6 mg kg^−1^) had a certain effect on the metabolic homeostasis of piglets.

Intestinal microbes are involved in protein metabolism, and three transcription factors involved in the regulation of methionine metabolism were identified in *Streptococcus* (Kovaleva and Gelfand 2007), suggesting that *Streptococcus* can participate in methionine metabolism; this partially explains the negative correlation between *Streptococcus* and the methionine metabolism pathway (Fig. 8). The relative abundance of *Streptococcus* was increased and methionine was decreased in fecal samples from the HC (300 mg kg^−1^) group, possibly because *Streptococcus* degrades the nitrogen compounds (methionine) in feed into ammonia (Varel et al. 1987), which confirms the negative correlation between *Streptococcus* and methionine (Fig. 7). Therefore, the decreased nitrogen compounds in piglet feces from the HC (300 mg kg^−1^) group and increased protein digestibility could also be due to an increase in the relative abundance of certain bacteria, accelerating the use of undigested nitrogenous compounds in the hindgut of piglets.

The present study investigated the microbiome-metabolome response in the hindgut of piglets fed different copper level diets. We found that consumption of diets with different copper levels differentially altered the microbial community composition and metabolic pathways in the hindgut, thus affecting the health of piglets receiving those diets. These alterations may help us assess the safety and necessity of excess Cu supplementation in feed and elucidate the impacts of copper intake on the health of animals and humans.

## Electronic supplementary material

ESM 1 (PDF 1422 kb)
