# Supplementary material for: Suhuai suckling piglet hindgut microbiome-metabolome responses to different dietary copper levels
Source: Appl Microbiol Biotechnol. 2018 Dec 7;103(2):853–68. doi: 10.1007/s00253-018-9533-0 (PMC6373200; doi:10.1007/s00253-018-9533-0)
Supplement: Supplementary file 1 — (PDF 1422 kb) [file 253_2018_9533_MOESM1_ESM.pdf]

Journal name: *Applied Microbiology and Biotechnology*

Suhuai suckling piglet hindgut microbiome-metabolome responses to different dietary copper levels

Feng Zhang[1, 2], Weijiang Zheng[1], Yongqiang Xue[1], Wen Yao[1, 3*]

[1]Jiangsu Key Laboratory of Gastrointestinal Nutrition and Animal Health, College of Animal Science and Technology, Nanjing Agricultural University, Nanjing, China

[2]College of Agriculture, Guangdong Ocean University, Zhanjiang, China

[3]Key lab of Animal Physiology and Biochemistry, Ministry of Agriculture, Nanjing, China

[*]**Correspondence:** Prof. Wen Yao, Email: yaowen67jp@njau.edu.cn

Supplementary Figures

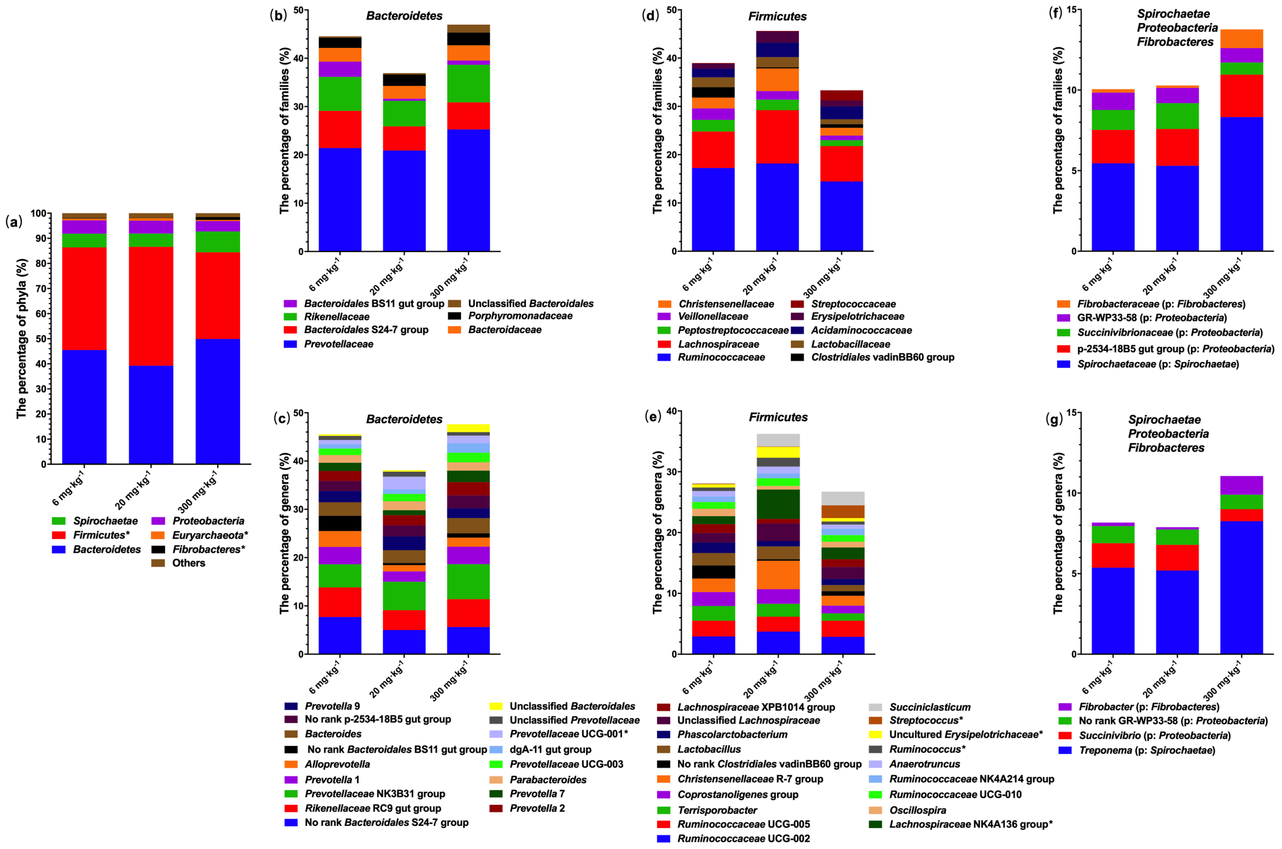

Fig. S1 The composition of fecal microbiota in suckling piglets at phylum, family, and genus level (relative abundance of more than 1%). The *Bacteroidetes*, *Firmicutes*, *Spirochaetae*, and *Proteobacteria* phyla constituted approximately 96% of the identified sequences (**a**), broken down as follows: seven families (**b**) and 17 genera (**c**) in *Bacteroidetes*; ten families (**d**) and 19 genera (**e**) in *Firmicutes*; five families (**f**) and four genera (**g**) in *Spirochaetae*, *Proteobacteria* and *Fibrobacteres*

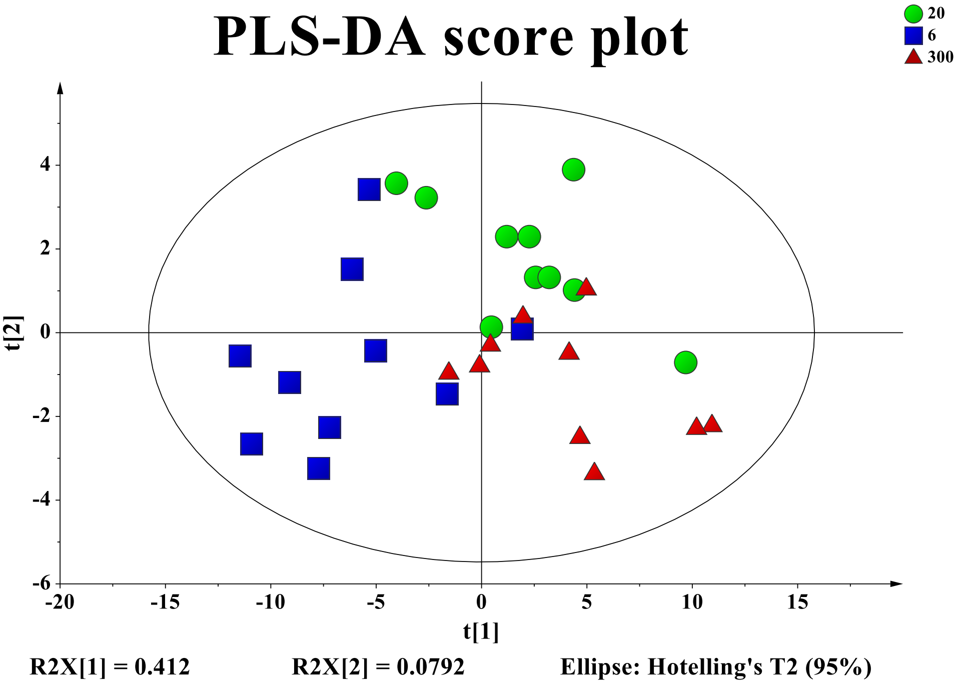

Fig. S2 Partial least squares-discriminant analysis (PLS-DA) of fecal metabolites in suckling piglets among each dietary copper group

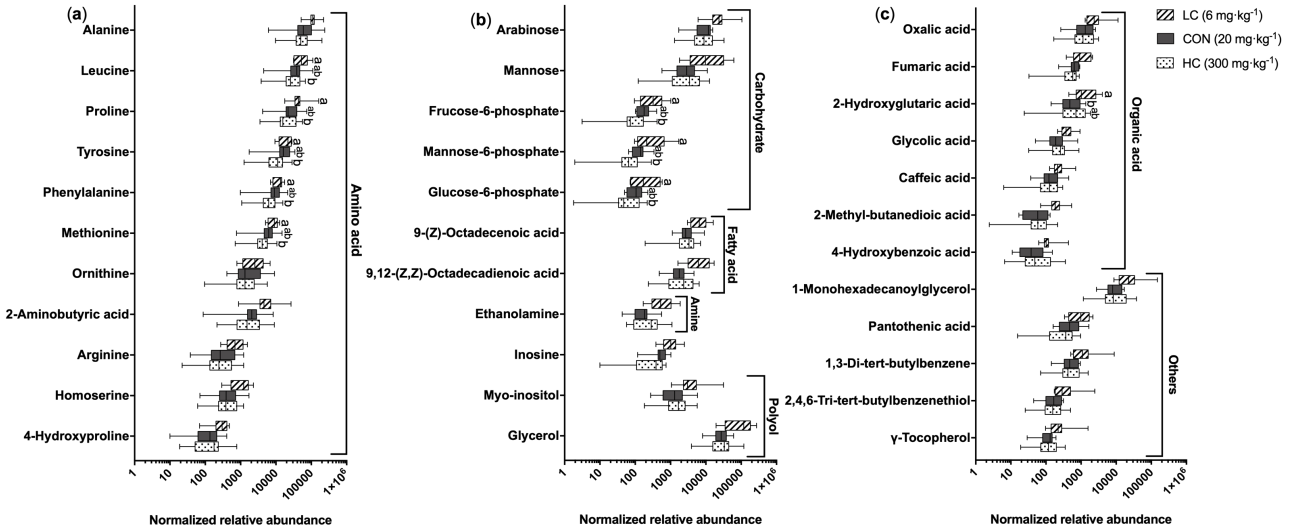

Fig. S3 Effect of dietary copper level on normalized relative abundance of fecal metabolites in suckling piglets

Supplementary Tables

Table S1. Significant compounds in fecal samples among each group of suckling piglets

Category	Metabolite	VIP[1]	*P*-value
Carbohydrate	Arabinose	1.41	<0.01
	Glucose	1.18	<0.01
	Mannose-6-phosphate	1.17	<0.01
	Mannose	1.16	<0.01
	Frucose-6-phosphate	1.15	<0.01
	Glucose-6-phosphate	1.14	<0.01
Amino acid	Lysine	1.51	<0.01
	Methionine	1.40	<0.01
	Alanine	1.39	<0.01
	Proline	1.34	<0.01
	Phenylalanine	1.30	<0.01
	Arginine	1.29	<0.01
	Valine	1.28	<0.01
	4-Hydroxyproline	1.24	<0.01
	Leucine	1.22	<0.01
	Ornithine	1.21	<0.01
	Homoserine	1.19	<0.01
	2-Aminobutyric acid	1.19	<0.01
	Isoleucine	1.18	<0.01
	Serine	1.14	<0.01
	Tyrosine	1.08	<0.01
	Aspartic acid	1.07	<0.05
	Threonine	1.02	<0.05
Fatty acid	9,12-(Z,Z)-Octadecadienoic acid	1.31	<0.01
	9-(Z)-Octadecenoic acid	1.19	<0.01
Amine	Putrescine	1.36	<0.01
	Ethanolamine	1.16	<0.01
Polyol	Glycerol	1.29	<0.01
	Myo-inositol	1.14	<0.01
Organic acid	2-Methyl-butanedioic acid	1.62	<0.01
	Caffeic acid	1.25	<0.01
	Glycolic acid	1.25	<0.01
	Malic acid	1.21	<0.05
	Fumaric acid	1.21	<0.05
	4-Hydroxybenzoic acid	1.11	<0.05
	Benzoic acid	1.10	<0.05
	Adipic acid	1.06	<0.05
	2-Hydroxyglutaric acid	1.06	<0.05

	Oxalic acid	1.03	<0.05
	Lactic acid	1.00	<0.05
Nucleotide	Inosine	1.28	<0.01
	Pseudouridine	1.14	<0.01
Others	Pantothenic acid	1.38	<0.01
	1,3-Di-tert-butylbenzene	1.38	<0.01
	Uracil	1.33	<0.01
	γ-Tocopherol	1.26	<0.01
	3-Hydroxypyridine	1.02	<0.05

[1]Variable important projection (VIP) value was obtained from partial least squares discriminant analysis (PLS-DA) model with a threshold of 1.0
